# Anti‐platelet antibody immunoassays in childhood immune thrombocytopenia: a systematic review

**DOI:** 10.1111/vox.12894

**Published:** 2020-02-20

**Authors:** David E. Schmidt, Anke J. Lakerveld, Katja M. J. Heitink‐Pollé, Marrie C. A. Bruin, Gestur Vidarsson, Leendert Porcelijn, Masja de Haas

**Affiliations:** ^1^ Sanquin Research Department of Experimental Immunohematology Amsterdam The Netherlands; ^2^ Landsteiner Laboratory Amsterdam UMC University of Amsterdam Amsterdam The Netherlands; ^3^ Department of Pediatric Hematology University Medical Center Utrecht Utrecht The Netherlands; ^4^ Princess Maxima Center for Pediatric Oncology Utrecht The Netherlands; ^5^ Department of Immunohematology Diagnostics Sanquin Diagnostic Services Amsterdam The Netherlands; ^6^ Sanquin Research Center for Clinical Transfusion Research Leiden The Netherlands; ^7^ Jon J van Rood Center for Clinical Transfusion Science Leiden University Medical Center Leiden The Netherlands; ^8^ Department of Immunohematology and Blood Transfusion Leiden University Medical Center The Netherlands

**Keywords:** immune thrombocytopenia, paediatrics, autoantibodies, clinical laboratory techniques, systematic review

## Abstract

**Background:**

In adult immune thrombocytopenia (ITP), an acquired autoimmune bleeding disorder, anti‐platelet autoantibody testing may be useful as a rule‐in test. Childhood ITP has different disease characteristics, and the diagnostic and prognostic value of anti‐platelet antibody testing remains uncertain.

**Objective:**

To systematically review the diagnostic accuracy of anti‐platelet autoantibody testing in childhood ITP.

**Methods:**

PubMed and EMBASE were searched for studies evaluating immunoassays in childhood ITP. Study quality was assessed (QUADAS2), and evidence was synthesized descriptively.

**Results:**

In total, 40 studies (1606 patients) were identified. Nine studies reported sufficient data to determine diagnostic accuracy measures. Anti‐platelet IgG antibody testing showed a moderate sensitivity (0·36–0·80 platelet‐associated IgG [direct test]; 0·19–0·39 circulating IgG [indirect test]). In studies that reported control data, including patients with non‐immune thrombocytopenia, specificity was very good (0·80–1·00). Glycoprotein‐specific immunoassays showed comparable sensitivity (three studies) and predominantly identified IgG anti‐GP IIb/IIIa antibodies, with few IgG anti‐GP Ib/IX antibodies. Anti‐platelet IgM antibodies were identified in a substantial proportion of children (sensitivity 0·62–0·64 for direct and indirect tests).

**Conclusion:**

The diagnostic evaluation of IgG and IgM anti‐platelet antibodies may be useful as a rule‐in test for ITP. In children with insufficient platelets for a direct test, indirect tests may be performed instead. A negative test does not rule out the diagnosis of ITP. Future studies should evaluate the value of anti‐platelet antibody tests in thrombocytopenic children with suspected ITP.

## Introduction

Childhood immune thrombocytopenia (ITP) is an autoimmune bleeding disorder 1. Given a lack of laboratory tests to rule‐in or rule‐out the diagnosis of ITP, the disease is diagnosed clinically by exclusion of alternative causes of thrombocytopenia 2, 3. This may lead to misdiagnoses, as much as 12% in adult ITP 4, for example for patients with hereditary platelet and immune disorders 5, 6. Misdiagnosed patients are not only exposed to unsuitable ITP‐specific treatments, but also the treatment‐associated side‐effects and costs. Misdiagnoses also lead to delayed diagnosis and adequate management of the actual underlying disorder. On the other hand, with new treatment options, for example the early administration of TPO agonists that are currently being investigated 7, clinicians may wish to ascertain an ITP diagnosis when considering such treatments. Thus, there is an unmet clinical need for laboratory tests to support the diagnosis of ITP 2.

A pathophysiological hallmark of ITP are anti‐platelet autoantibodies specific to platelet glycoproteins 8, 9. Soon after the discovery of these platelet autoantibodies, assays to measure anti‐platelet antibodies were developed 10, 11 and several studies identified anti‐platelet antibodies in childhood ITP 11, 12, 13. Nonetheless, anti‐platelet antibody testing currently remains of unclear clinical benefit 2, 3, for several reasons. The results of early studies were dismissed by the observation of non‐specific adsorption of plasma protein to platelets and potential false‐positive results in healthy controls and patients with non‐immune thrombocytopenia 14, 15, 16, 17, 18, 19, 20. Later, to overcome these challenges, antigen‐specific assays were developed that are now considered standard for assessment of anti‐platelet antibodies in ITP 16, 21, 22. A recent systematic review suggested that such antigen‐specific anti‐platelet antibody testing may be a useful rule‐in test for adult ITP 23. However, it is unknown whether this finding is transferable for childhood ITP. In contrast to adult ITP, childhood ITP has a large proportion of patients with preceding infections and mostly self‐limiting disease courses, and the clinical context of suspected ITP in children is clearly different. A further reason for uncertainty is the heterogeneous background of ITP, which represents a mix of cases with distinct underlying pathophysiology. For instance, ITP may also be caused by T‐cell‐mediated immunity directed towards platelet autoantigens and megakaryocytes 24. It is unknown how this influences the diagnostic and prognostic value of anti‐platelet antibody testing. In sum, the role of anti‐platelet antibody testing in the diagnosis of childhood ITP remains uncertain.

In the present study, we aimed to synthesize the available evidence to determine the potential diagnostic accuracy of anti‐platelet antibody testing in childhood ITP. In addition, we describe some data on the prognostic significance of anti‐platelet antibody testing.

## Methods

### Study identification and quality assessment

**Figure 1:**
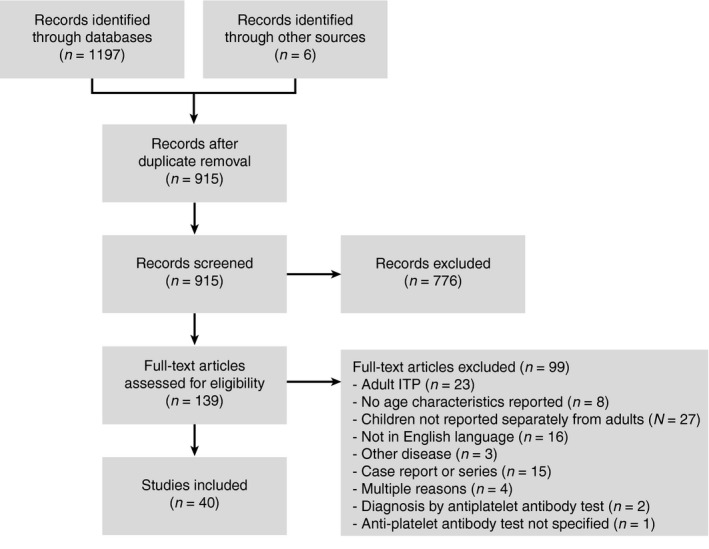
Flowchart of study identification and assessment.

Reporting standards of the PRISMA guidelines were followed. PubMed and EMBASE were searched from inception until 4 April 2019 to determine the diagnostic accuracy of various immunoassays measuring platelet autoantibodies in childhood ITP (Table S1). The search string contained three elements: domain (children, 3 months to 18 years), disease (ITP) and diagnostic tests for comparison (platelet autoantibody tests). The sensitivity of the search strategy was assessed by the inclusion of pre‐determined index publications. Screening of abstracts, full‐text assessment and data extraction was performed independently by two investigators (D.S. and A.L.; Fig. 1). Studies were included if they were published in English language and evaluated a platelet autoantibody assay in children with immune thrombocytopenia and fully described the anti‐platelet antibody immunoassay in the manuscript or a previous publication. Studies were excluded if they did not separate adult and childhood ITP data, assessed neonates or used anti‐platelet antibodies as criteria for the diagnosis of ITP. Case series (less than 10 patients) were excluded. Data collection was validated independently by a third investigator (L.P.). The methodological quality of included articles was assessed using a standardized protocol for quality assessment of diagnostic accuracy studies (QUADAS2) 25.

Methodological details are described in the Supplementary Methods.

### Synthesis of results

Given substantive heterogeneity in study populations and methodological quality, study results were not pooled for meta‐analysis of diagnostic accuracy.

## Results

### Study identification and characteristics

After screening and full‐text assessment, 40 studies (1606 patients) were identified for this systematic review (Fig. 1; Table S2). The studies assessed either platelet‐associated antibodies on autologous patient platelets (direct test) or circulating anti‐platelet antibodies (indirect test).

### Assessment of risk of bias and concerns for applicability

**Figure 2:**
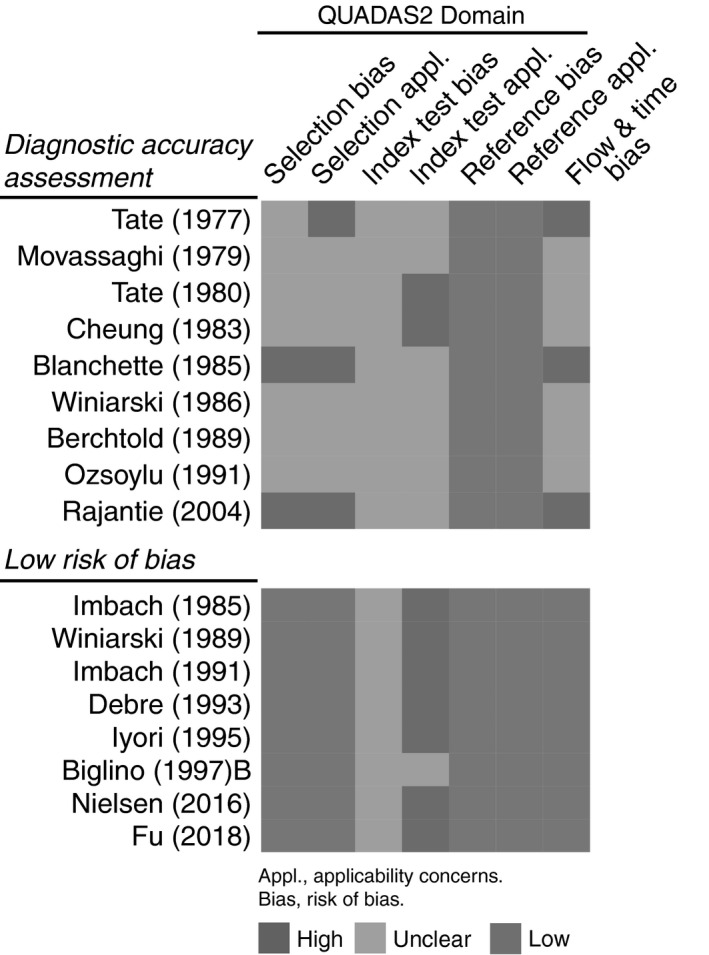
Quality assessment of studies with available data for diagnostic accuracy assessment, as well as studies including patients with a low risk of bias, with judgement presented per study (full assessment of 40 studies in Fig. S1).

We first assessed the methodological quality of included studies across four domains: (1) recruitment and inclusion of patients (patient selection), (2) the evaluated anti‐platelet antibody test (index test), (3) the criteria for diagnosis of ITP (reference standard) and (4) included disease stages and treatments (flow and timing). Overall, this assessment indicated that the risk of bias was unclear or high for a large proportion of studies (Fig. S1). Three factors were primarily responsible for this. First, although studies usually assessed the assay in at least some controls (either in the same study or historically), a substantial analysis was often lacking for control subjects, or this was incompletely reported. Second, studies did not report sensitivity and specificity or dichotomized test results from which these could be derived, that is true‐positive, true‐negative, false‐negative and false‐positive results. Third, many studies included heterogeneous disease stages with varying time from diagnosis, as well as mixing patients with prior or current treatments, both of which are known to influence anti‐platelet antibody testing 11, 13, 26. Nine of the 40 studies reported sufficient data to determine diagnostic accuracy of anti‐platelet antibody testing; all nine were of unclear or high risk of bias (Fig. 2; top panel) 27, 28, 29, 30, 31, 32, 33, 34, 35. Eight of the 40 included studies were judged to be of low risk of bias regarding the inclusion of ITP patients, but none of them explicitly reported control data (Fig. 2; lower panel) 36, 37, 38, 39, 40, 41, 42, 43. We subsequently focused our analysis on the nine studies which allowed assessment of diagnostic accuracy data and describe data of the eight studies with low risk of bias and the remaining studies, as applicable.

### Diagnostic accuracy of anti‐platelet antibody testing in childhood ITP

Nine studies allowed an assessment of the diagnostic accuracy of anti‐platelet antibody testing (Table 1). The studies had a median sample size for cases and controls of 21 and 20, respectively. When we assessed these studies together, the primary finding was that most of the nine studies showed moderate sensitivity of immunoassays, with very good to excellent specificity (Table 2). These results were consistent across small study cohorts (N < 20) and larger studies, as well as amongst studies that included vs. did not include non‐immune thrombocytopenic controls. Indirect tests potentially have a lower sensitivity than direct tests, but a direct comparison was lacking (Table 2). Specificity was high across the studies (Table 2). Importantly, although false‐positive results may be missed by small studies, specificity results were the same in studies which included small or large control groups. Next, we assessed some of the methodological concerns of these studies with unclear or high risk of bias. Treatment with steroids (two studies) may have lowered the sensitivity of the tests; one small study included IVIg‐treated patients that may lead to false‐positive test results 33, and another study measured anti‐platelet antibody levels in IVIg‐treated patients, but by an antigen‐specific immunobead assay 35. The inclusion of patients later in the disease course, as opposed to assessment at diagnosis, may skew results, but the studies at low risk of bias which included patients within a week of diagnosis showed a similar sensitivity (discussed below).

**Table 1:** Characteristics of included studies (selection)

Author	Year	Region	Study design	Immunoassay class	Sample size	Age (years)	Female (%)	Chronic ITP (%)	Prior and current treatments
Studies for assessment of diagnostic accuracy									
Tate	1977	USA	Unclear	PA‐Ig	10	6·8 (mean)	40	70	Steroids (4/10)
Movassaghi	1979	USA	Unclear	Functional	42	5·3 (1·25–14; median, range)	61	nd	Treatment naive
Tate	1980	USA	Unclear	PA‐Ig	12^a^	1–14 (range)	25	nd	nd
Cheung	1983	USA	Prospective cohort	ELISA	48	0·25–16 (range)	nd	46	nd
Blanchette	1985	Canada	Prospective cohort	PA‐Ig	29	acute: 3 (0·25–16; median, range) chronic: 9 (3–17; median, range)	55	28	Steroids (11 of acute; 2 of chronic)
Winiarski	1986	Sweden	Prospective cohort	ELISA	18^c^	nd	39	0	nd
Berchtold	1989	USA	Prospective cohort	Immunobead	39	acute: 5 (0·6–11; median, range) chronic: 10 (3–19; median, range)	46	62	IVIg and corticosteroids
Ozsoylu	1991	Turkey	Unclear	Functional	149	0·25–15 (range)	nd	31	nd
Rajantie	2004	Finland	Prospective cohort	PIFT	13/14^b^	6·2 ± 1·1 (mean ± SE)	57	64	IVIg and steroids (6/14), steroids (2/14)
Studies with homogeneous patient populations and low risk of bias									
At diagnosis									
Imbach	1985	Switzerland, Germany	Clinical trial	PA‐Ig	57/94^b^	6 (mean)	52	37	Treatment naive
Winiarski	1989	Sweden	Prospective cohort	Immunoblot	21	1·5–15 (range)	29	nd	Treatment naive
Debre	1993	France	Clinical trial	PA‐Ig	10/12^b^	7·2 (3–13; mean, range)	42	nd	Treatment naive
Biglino	1997b	Italy	Unclear	MAIPA	74	5·5 (0·25–13; mean, range)	nd	34	Treatment naive
Nielsen	2016	Denmark	Retrospective cohort	PA‐Ig	68	44% is up to 3 years	56	18	Treatment naive
Fu	2018	China	Prospective cohort	ELISA	134	1·6 (0·1–13·25; mean, range)	43	13	Treatment naive
Chronic ITP									
Imbach	1991	Multinational	Prospective cohort	Immunobead	36	1–16 (range)	nd	100	No treatment 3 weeks before inclusion
Iyori	1995	Japan	Prospective cohort	PIFT, PA‐Ig	29	11 (1·8–21·2; mean, range)	38	100	Splenectomy (2/29); rest untreated

Full data of all 40 identified studies are given in the Supplementary Tables.

nd, not described.

a Two adults originally included in the study were excluded.

b Only *n*/N patients had anti‐platelet antibodies tested; characteristics are reported for the whole cohort.

c Children with chronic disease were not included as they were not distinguishable from adults.

**Table 2:** Diagnostic accuracy of anti‐platelet antibody testing

Author	Year	Immunoassay class	Test type	IgG class	Type ITP	Type Controls	ITP (N)	Positive (*n*)	Negative (*n*)	Controls (*n*)	Positive (*n*)	Negative (*n*)	Sensitivity (95% CI)	Specificity (95% CI)	Positive LR (95% CI)	Negative LR (95% CI)
Tate	1977	PA‐Ig	Indirect	Total Ig		Healthy controls; non‐immune thrombocytopenia	10	10	0	19	1	18	1·00 (nd)	0·95 (0·85; 1·00)	19·0 (2·8; 128)	nd
Movassaghi	1979	Functional	Indirect	Total Ig		Healthy controls; non‐immune thrombocytopenia	42	23	19	155	7	148	0·55 (0·40; 0·70)	0·96 (0·92; 0·99)	12·1 (5·6; 26·3)	0·47 (0·34; 0·66)
Tate	1980	PA‐Ig	Indirect	IgG		Healthy controls; non‐immune thrombocytopenia	12	12	0	27	0	27	1·00 (nd)	1·00 (nd)	nd	nd
Cheung	1983	ELISA^b^	Direct	IgG	Acute	Healthy controls	26	22	4	17^a^	0	17	0·85 (0·71; 0·98)	1·00 (nd)	nd	0·15 (0·06; 0·38)
Cheung	1983	ELISA^b^	Direct	IgG	Chronic	Healthy controls	22	22	0	17^a^	0	17	1·00 (nd)	1·00 (nd)	nd	nd
Blanchette	1985	PA‐Ig	Direct	IgG	Acute	Healthy children; non‐immune thrombocytopenia; non‐immune haematological disorders	21	16	5	46	4^a^	42	0·76 (0·58; 0·94)	0·91 (0·83; 0·99)	8·8 (3·3; 23·0)	0·26 (0·12; 0·56)
Blanchette	1985	PA‐Ig	Direct	IgG	Chronic	Healthy children; non‐immune thrombocytopenia; non‐immune haematological disorders	8	7	1	46	4^a^	42	0·88 (0·65; 1·00)	0·91 (0·83; 0·99)	10·1 (3·8; 26·6)	0·14 (0·02; 0·86)
Winiarski	1986	ELISA	Indirect	IgG		Healthy blood donors	21^c^	10	11	25	1	24	0·48 (0·26; 0·69)	0·96 (0·88; 1·00)	11·9 (1·7; 85·5)	0·55 (0·36; 0·83)
Berchtold	1989	Immunobead, GP IIb/IIIa	Indirect	IgG	Acute	Healthy children	15	4	11	10	0	10	0·27 (0·05; 0·49)	1·00 (nd)	nd	0·73 (0·54; 0·99)
Berchtold	1989	Immunobead, GP IIb/IIIa	Indirect	IgG	Chronic	Healthy children	24	14	10	10	0	10	0·58 (0·38; 0·78)	1·00 (nd)	nd	0·42 (0·26; 0·67)
Berchtold	1989	Immunobead, GP Ib/IX	Indirect	IgG	Acute	Healthy children	15	0	15	10	0	10	0·00 (nd)	1·00 (nd)	nd	nd
Berchtold	1989	Immunobead, GP Ib/IX	Indirect	IgG	Chronic	Healthy children	24	0	24	10	0	10	0·00 (nd)	1·00 (nd)	nd	nd
Ozsoylu	1991	Functional	Indirect	Total Ig		Healthy children; non‐immune thrombocytopenia	146	146	0	126	0	126	1·00 (nd)	1·00 (nd)	nd	nd
Rajantie	2004	PIFT	Direct	Total Ig		Healthy children; familal thrombocytopenia	13	7	6	10	2	8	0·54 (0·27; 0·81)	0·80 (0·55; 1·00)	2·7 (0·7; 10·3)	0·58 (0.30; 1.12)

Only studies were included that presented dichotomized results or quantitative data in a graph.

CI, confidence interval; LR, likelihood ratio.

a Number abstracted from graph.

b The employed assay involved radiochemistry instead of enzyme, but the test principle falls under the ELISA category.

c 21 sera tested from 18 patients. nd, not determined.

We calculated positive likelihood ratios that indicate how much more likely it is that a positive test result occurs in a patient, compared to controls, where ratios of ≥10 are considered clinically useful to rule‐in disease 44. Amongst four of the nine studies (92 patients) where positive likelihood ratios (LR) could be calculated, the positive LR was at least 8·8 in three studies, indicating that the assays could be used as rule‐in tests for childhood ITP. In the other studies, only patients showed positive test results. On the other hand, with the given sensitivity and negative LR ranging between 0·14 and 0·73 (170 patients), the assays were insufficient as a rule‐out criterion for ITP, for which a negative LR of ≤0·1 would be considered useful. In sum, there was weak‐to‐moderate evidence that anti‐platelet antibody testing could potentially be useful as a rule‐in test of ITP.

### Supporting evidence

We next compared these data to results of the other studies that were identified by our systematic review. First, amongst the eight studies with low risk of bias (Table 1), six studies assessed anti‐platelet IgG in patients with newly diagnosed ITP within a week of diagnosis, and two studies included patients with chronic ITP (Table 3; results of all 40 identified studies are shown in Table S5). We observed a similar moderate sensitivity as for the studies discussed above. Moreover, sensitivity was the same for newly diagnosed, as well as chronic ITP, and for glycoprotein‐specific assays 39, 40, 42, 43. The results were unchanged when only the largest studies were considered 37, 42, 43.

**Table 3:** Sensitivity of anti‐platelet antibody testing in ITP (studies with low risk of bias)

Author	Year	Immunoassay class	Type test	Subclass	Antigen	Prognosis	ITP (N)	Positive (*n*)	Negative (*n*)	Sensitivity	95% CI
At diagnosis											
Imbach	1985	PA‐Ig	Direct	IgG			57	43	14	0·75	0·64; 0·87
Debre	1993	PA‐Ig	Direct	IgG			10	8	2	0·80	0·55; 1·00
Nielsen	2016	PA‐Ig	Direct	IgG			68	30	38	0·44	0·32; 0·56
Nielsen	2016	PA‐Ig	Direct	IgM			68	43	25	0·63	0·52; 0·75
Winiarski	1989	Immunoblot	Indirect	IgG			21	4	17	0·19	0·02; 0·36
Winiarski	1989	Immunoblot	Indirect	IgM			21	13	8	0·62	0·41; 0·83
Biglino	1997b	MAIPA	Indirect	IgG	GP IIb/IIIa	Transient	49	19	30	0·39	0·25; 0·52
Biglino	1997b	MAIPA	Indirect	IgG	GP IIb/IIIa	Chronic	25	8	17	0·32	0·14; 0·50
Biglino	1997b	MAIPA	Indirect	IgG	GP Ib/IX	Transient	49	15	34	0·31	0·18; 0·44
Biglino	1997b	MAIPA	Indirect	IgG	GP Ib/IX	Chronic	25	7	18	0·28	0·10; 0·46
Fu	2018	ELISA	Indirect	IgG	GP IIb/IIIa	Transient	113	92	21	0·81	0·74; 0·89
Fu	2018	ELISA	Indirect	IgG	GP IIb/IIIa	Chronic	18	11	7	0·61	0·39; 0·84
Fu	2018	ELISA	Indirect	IgG	GP Ib/IX	Transient	113	54	59	0·48	0·39; 0·57
Fu	2018	ELISA	Indirect	IgG	GP Ib/IX	Chronic	18	11	7	0·61	0·39; 0·84
Chronic ITP											
Imbach	1991	Immunobead	Direct	IgG	GP IIb/IIIa, GP Ib^b^		36	26	10	0·72	0·58; 0·87
Iyori	1995	PA‐Ig	Direct	IgG	GP IIb/IIIa^a^		25	9	16	0·36	0·17; 0·55
Iyori	1995	PIFT	Direct	IgG			29	22	7	0·76	0·60; 0·91
Iyori	1995	PIFT	Direct	IgM			29	20	9	0·69	0·52; 0·86

a By immunoprecipitation of the antigen in platelet lysates.

b Results were reported only in aggregate.

For the remainder of the studies, a further eight reported quantitative results (i.e. mean or median and distribution) of platelet antibody immunoassays in cases and controls (Table S3). These eight studies unanimously indicated distinct anti‐platelet antibody levels in patients with ITP compared to controls (Table S3). Thus, even though it was not possible to calculate the diagnostic accuracy, these studies confirmed that anti‐platelet antibody levels are elevated in ITP patients compared to controls.

Together, these findings provide support that IgG anti‐platelet antibodies can be detected in children with ITP and that results could be relatively specific for ITP.

### Detection of anti‐platelet IgM and IgA antibodies

Three of the eight studies with low risk of bias assessed immunoassays for evaluation of anti‐platelet IgM antibodies, two in newly diagnosed ITP and one in chronic ITP (Table 3; full results, Table S6). The sensitivity for detection of IgM autoantibodies was 0·62–0·69, indicating that IgM class autoantibodies could potentially be as prevalent as anti‐platelet IgG antibodies 37, 39, 41. Due to a lack of control data, the specificity could not be determined. Nonetheless, these findings in studies with low risk of bias suggest that IgM anti‐platelet antibodies could be of importance in childhood ITP.

Three studies assessed the presence of anti‐platelet IgA antibodies using direct ELISA or direct and indirect PIFT, identifying such antibodies in 5–24% of patients (Table S7). In comparison with IgM and IgG autoantibodies assessed in the same study by the same assay, IgA was detected at a 2‐ to 10‐fold lower frequency 12, 45, 46. None of these studies was of low risk of bias. Thus, IgA antibodies might be implicated in ITP, but a more extensive assessment is required to determine their role for diagnostic testing.

### Association of anti‐platelet antibodies with prognosis

Some of the included studies assessed the association of anti‐platelet antibodies in early disease with future disease outcomes. Nielsen *et al.* observed that only 7% of patients with chronic disease course showed glycoprotein‐specific IgM or IgG antibodies, compared to 41% of patients with a transient ITP course (N = 15 vs. N = 37) 47. Moreover, when measuring antibodies at diagnosis, Fu *et al.* found that patients with a prognosis of chronic ITP showed enrichment of anti‐GP Ib/IX antibodies, compared to those with transient disease courses (N = 18 vs. N = 113) 43. Findings of these two studies are contrasted by Biglino *et al.* who observed equal rates of anti‐GPIIb/IIIa or anti‐GPIb/IX antibodies in patients with transient or chronic disease courses (N = 49 vs. N = 25)^42^ and Nielsen *et al.* who showed a similar rate of anti‐platelet antibodies by PA‐IgG/IgM amongst patients with transient and chronic diseases courses (N = 46 vs. N* = *12)^37^. Taken together, some studies indicate a role of anti‐platelet antibody testing to determine prognosis, but in the presence of conflicting evidence further data are required.

## Discussion

In this study, we systematically reviewed 40 studies and summarized the current knowledge regarding the diagnostic accuracy of anti‐platelet antibody testing in childhood ITP. Although many studies suffered from insufficient reporting and a lack of controls, we carefully analysed and weighted the available data. The main finding of this review is the overall good diagnostic accuracy of anti‐platelet antibody testing, based on multiple case–control and prospective cohort studies, supported by evidence from low risk of bias studies. Sensitivity was moderate in multiple studies, and this could be due to low levels of circulating antibodies during active clearance of platelet‐antibody immune complexes, low avidity of the antibodies or insensitivity of the used tests or heterogeneity in the involved pathomechanisms, such as antibody‐mediated or T‐cell‐mediated anti‐platelet clearance 24. Even though evidence has been published that indicated potential false‐positive results in patients with thrombocytopenia by other causes than ITP, particularly with PA‐Ig assays 14, 15, 16, studies included in this review showed good specificity in healthy children and patients with non‐immune thrombocytopenia (multiple case–control studies), also in the largest included studies 28, 31, 32. It might be argued that studies that did not report control data showed a high rate of false‐positive results. Regardless, studies which used the more recently developed antigen‐specific assays 16 showed similar sensitivity as whole‐platelet testing (three studies; two with low risk of bias), which provides a compelling argument against skewing to false‐positive results. Thus, when balancing the available data on diagnostic accuracy, anti‐platelet antibody assays may potentially be used as a rule‐in test, as also indicated by a high positive likelihood ratio. This is in line with results of a recent systematic review of anti‐platelet antibody testing in adult ITP 23. On the other hand, given the limited sensitivity, the data show that anti‐platelet antibody assays cannot be used as a rule‐out test to exclude a diagnosis of ITP, which is also in agreement with the conclusions of the systematic review in adult ITP 23.

A secondary finding of our analysis in childhood ITP was that IgM anti‐platelet antibodies may be as prevalent as IgG anti‐platelet antibodies (three studies), which should be assessed in future studies. Moreover, tests for circulating anti‐platelet antibodies are interesting alternatives when children have insufficient platelets available for a direct test. We found that although indirect tests might have a reduced sensitivity, they could still be of diagnostic value. Several studies described lower anti‐platelet antibody levels when patients were assessed later in their disease course or after treatment 11, 13, 28, 32, 48, indicating that delayed testing may affect the diagnostic accuracy. Finally, with regard to relevant platelet antigens, in two studies, anti‐GPIb/IX antibodies were not detected at all, and GP IIb/IIIa was the predominant anti‐platelet antibody 35, 49. Conversely, antibodies directed against GP IIb/IIIa and GP Ib/IX were found at about the same rate by one study 43. Thus, the role of antibodies against specific platelet glycoproteins for the diagnosis and prognosis of childhood ITP remains elusive.

The primary limitation of this review was the reference test of our study (gold standard), which was a clinical diagnosis of ITP. As surrogates of patient heterogeneity between studies, we extracted data on age, sex and preceding infection, which are associated with ITP prognosis 50. Moreover, the reported diagnostic accuracy may be optimistic estimates, since none of the studies investigated anti‐platelet antibody assays in children with suspected ITP. However, in the context of a rare disease and a paediatric study population, such studies are notoriously difficult to conduct. Two further shortcomings regarding the methodological quality of included studies limited our review: (1) insufficient reporting of control data to determine specificity for the majority of studies and (2) the inclusion of heterogeneous patient populations at various disease stages and divergent treatments. Nonetheless, we recognized that the identified studies represent the best evidence to date in a disease setting that is challenging to investigate, and carefully assessed and compared the available data. We suggest that to improve the quality of data in the field, future studies published in childhood ITP should be explicitly required to disclose clinical characteristics, inclusion criteria, time from diagnosis and previous and current treatments.

Finally, during routine diagnostic testing, not all childhood patients have sufficient platelet numbers to perform direct tests; thus, results could be biased to older children, for whom more material is available. None of the studies disclosed the number of patients that could not be evaluated.

For future directions, direct tests should be developed that allow the use of low number of platelets. The assessment of anti‐platelet antibodies may be of prognostic significance, potentially indicating a subgroup amongst heterogenous ITP patients, and this should be investigated further. A key current area of uncertainty is the evaluation of anti‐platelet antibody assays in children with suspected ITP.

In conclusion, this systematic review indicates that anti‐platelet antibody testing could potentially be used as a rule‐in test for childhood ITP, although anti‐platelet antibody testing cannot be used to exclude a diagnosis of ITP.

## Conflict of interest

The authors declare no competing financial interests.

## Author contributions

D.E.S. and A.J.L. screened and assessed articles. A.J.L. wrote an initial draft. D.E.S. wrote the manuscript. L.P. assessed articles, validated and interpreted results. K.M.J.H.‐P., M.C.A.B. and G.V. interpreted results. M.d.H. supervised the study. All authors revised and approved the manuscript.

## Supporting information


**Fig. S1** Quality assessment of all 40 included studies, with judgement presented per study.

**Table S1** Search strategy for Pubmed and EMBASE searching.
**Table S2** Study characteristics of 40 included studies.
**Table S3** Quantitative differences of anti‐platelet antibody levels in ITP versus controls.
**Table S4** Sensitivity of anti‐platelet antibody testing in ITP for total Ig.
**Table S5** Sensitivity of anti‐platelet antibody testing in ITP for IgG isotype.
**Table S6** Sensitivity of anti‐platelet antibody testing in ITP for IgM isotype.
**Table S7** Sensitivity of anti‐platelet antibody testing in ITP for IgA and unclear Ig target.

## References

[1] Terrell DR , Beebe LA , Vesely SK , *et al*: The incidence of immune thrombocytopenic purpura in children and adults: A critical review of published reports. Am J Hematol. 2010; 85:174–80 20131303 10.1002/ajh.21616

[2] Provan D , Stasi R , Newland AC , *et al*: International consensus report on the investigation and management of primary immune thrombocytopenia. Blood. 2010; 115:168‐86 19846889 10.1182/blood-2009-06-225565

[3] Neunert C , Lim W , Crowther M , *et al*: The American Society of Hematology 2011 evidence‐based practice guideline for immune thrombocytopenia. Blood 2011; 117:4190–4207 21325604 10.1182/blood-2010-08-302984

[4] Arnold DM , Nazy I , Clare R , *et al*: Misdiagnosis of primary immune thrombocytopenia and frequency of bleeding: lessons from the McMaster ITP Registry. Blood Advances 2017; 1:2414–2420 29296891 10.1182/bloodadvances.2017010942 PMC5729626

[5] Althaus K , Najm J , Greinacher A : MYH9 related platelet disorders ‐ often unknown and misdiagnosed. Klin Padiatr 2011; 223:120–5 21567368 10.1055/s-0031-1275664

[6] Bader‐Meunier B , Proulle V , Trichet C , *et al*: Misdiagnosis of Chronic Thrombocytopenia in Childhood. J Pediatr Hematol Oncol 2003; 25:548–552. B. Bader‐Meunier, Department of Pediatrics, Bicetre Hospital, 94270 Le Kremlin Bicetre, France. E‐mail: brigitte.bader‐meunier@bct.ap‐hop‐paris.fr: Lippincott Williams and Wilkins (530 Walnut Street, P O Box 327, Philadelphia PA 19106–3621, United States). 12847322 10.1097/00043426-200307000-00010

[7] Eltrombopag vs Standard Front Line Management for Newly Diagnosed Immune Thrombocytopenia (ITP) in Children [Internet]. Clinicaltrials.gov. [cited 2019 Oct 25]. Available from: https://clinicaltrials.gov/ct2/show/NCT03939637.

[8] van Leeuwen EF , van der Ven JT , Engelfriet CP , *et al*: Specificity of autoantibodies in autoimmune thrombocytopenia. Blood 1982; 59:23–6 7032627

[9] Shulman NR , Marder VJ , Weinrach RS : Similarities between known antiplatelet antibodies and the factor responsible for thrombocytopenia in idiopathic purpura. physiologic, serologic and isotopic studies. Ann N Y Acad Sci 1965; 124:499–542 5214832 10.1111/j.1749-6632.1965.tb18984.x

[10] McMillan R , Smith RS , Longmire RL , *et al*: Immunoglobulins associated with human platelets. Blood 1971; 37:316–22 5542152

[11] Cines DB , Schreiber AD : Immune Thrombocytopenia. N Engl J Med 1979; 300:106–111. 569254 10.1056/NEJM197901183000302

[12] van Leeuwen EF , von dem Borne AE , van der Plas‐van Dalen C , *et al*: Idiopathic thrombocytopenic purpura in children; detection of platelet autoantibodies by immunofluorescence. Scand J Haematol. 1981; 26:285–91 7038843 10.1111/j.1600-0609.1981.tb01663.x

[13] Lightsey ALJ , Koenig HM , McMillan R , *et al*: Platelet‐associated immunoglobulin G in childhood idiopathic thrombocytopenic purpura. J Pediatr 1979; 94:201–4 570218 10.1016/s0022-3476(79)80823-9

[14] Mueller‐Eckhardt C , Kayser W , Mersch‐Baumert K , *et al*: The clinical significance of platelet‐associated IgG: a study on 298 patients with various disorders. Br J Haematol 1980; 46:123–31 7191717 10.1111/j.1365-2141.1980.tb05942.x

[15] Kelton JG , Steeves K : The amount of platelet‐bound albumin parallels the amount of IgG on washed platelets from patients with immune thrombocytopenia. Blood 1983; 62:924–7 6683982

[16] Warner MN , Moore JC , Warkentin TE , *et al*: A prospective study of protein‐specific assays used to investigate idiopathic thrombocytopenic purpura. Br J Haematol 1999; 104:442–7 10086776 10.1046/j.1365-2141.1999.01218.x

[17] Borne AEGK , Verheugt FWA , Oosterhof F , *et al*: A simple immunofluorescence test for the detection of platelet antibodies. Br J Haematol 1978; 39:195–207 354684 10.1111/j.1365-2141.1978.tb01089.x

[18] Bizzaro N , Brandalise M : EDTA‐dependent pseudothrombocytopenia. Association with antiplatelet and antiphospholipid antibodies. Am J Clin Pathol. 1995; 103:103–7 7817934 10.1093/ajcp/103.1.103

[19] Muniz‐Diaz E , Madoz P , Pujol‐Moix N , *et al*: Characterization of antibodies directed against platelet cryptantigens detected during the immunological study of 356 consecutive patients with presumed autoimmune thrombocytopenia (AITP). Transfus Med (Oxford, England). 1995; 5:185–91 10.1111/j.1365-3148.1995.tb00226.x 8593522

[20] Kelton JG , Murphy WG , Lucarelli A , *et al*: A prospective comparison of four techniques for measuring platelet‐associated IgG. Br J Haematol 1989; 71:97–105 2917134 10.1111/j.1365-2141.1989.tb06281.x

[21] Kiefel V , Santoso S , Weisheit M , *et al*: Monoclonal antibody–specific immobilization of platelet antigens (MAIPA): a new tool for the identification of platelet‐reactive antibodies. Blood 1987; 70:1722–6 2445398

[22] Arnold DM , Santoso S , Greinacher A , *et al*: Recommendations for the implementation of platelet autoantibody testing in clinical trials of immune thrombocytopenia. J Thromb Haemost 2012; 10:695–697 22332994 10.1111/j.1538-7836.2012.04664.x PMC4854629

[23] Vrbensky JR , Moore JE , Arnold DM , *et al*: The sensitivity and specificity of platelet autoantibody testing in immune thrombocytopenia: a systematic review and meta‐analysis of a diagnostic test. J Thromb Haemost 2019; 17:787–94 30801909 10.1111/jth.14419

[24] Zufferey A , Kapur R , Semple JW : Pathogenesis and therapeutic mechanisms in immune thrombocytopenia (ITP). J Clin Med 2017; 6:16

[25] Whiting PF , Rutjes AWS , Westwood ME , *et al*: QUADAS‐2: a revised tool for the quality assessment of diagnostic accuracy studies. Ann Intern Med 2011; 155:529–36 22007046 10.7326/0003-4819-155-8-201110180-00009

[26] Ljung R , Nilsson IM , Frohm B , *et al*: Platelet‐associated IgG in childhood idiopathic thrombocytopenic purpura: measurements on intact and solubilized platelets and after gammaglobulin treatment. Scand J Haematol 1986; 36:402–7 2424081 10.1111/j.1600-0609.1986.tb01757.x

[27] Tate DY , Sorenson RL , Gerrard JM , *et al*: An immunoenzyme histochemical technique for the detection of platelet antibodies from the serum of patients with idiopathic (autoimmune) thrombocytopenic purpura (ITP). Br J Haematol 1977; 37:265–75 341958 10.1111/j.1365-2141.1977.tb06843.x

[28] Movassaghi N , Moorhead J , Leikin S : Antiplatelet antibodies in childhood idiopathic thrombocytopenic purpura. Am J Dis Child 1979; 133:257–9 570802 10.1001/archpedi.1979.02130030033003

[29] Tate DY , Carlton GT , Nesbit ME , *et al*: Detection of platelet associated IgG in immune thrombocytopenia: a new assay employing protein A and peroxidase anti‐peroxidase (PROA‐PAP). Am J Hematol 1980; 9:349–61 7011000 10.1002/ajh.2830090402

[30] Cheung NK , Hilgartner MW , Schulman I , *et al*: Platelet‐associated immunoglobulin G in childhood idiopathic thrombocytopenic purpura. J Pediatr 1983; 102:366–70 6681837 10.1016/s0022-3476(83)80650-7

[31] Blanchette V , Hogan V , Esseltine D , *et al*: Evaluation of a simple immunodiffusion technique for quantitation of platelet‐associated immunoglobulin G in childhood immune thrombocytopenias. Am J Pediatr Hematol Oncol 1985; 7:125–31 3939560

[32] Ozsoylu S , Karabent A , Irken G , *et al*: Antiplatelet antibodies in childhood idiopathic thrombocytopenic purpura. Am J Hematol 1991; 36:82–5 2012069 10.1002/ajh.2830360203

[33] Rajantie J , Javela K , Joutsi‐Korhonen L , *et al*: Chronic thrombocytopenia of childhood: use of non‐invasive methods in clinical evaluation. Eur J Haematol 2004; 72:268–72 15089765 10.1111/j.1600-0609.2004.00215.x

[34] Winiarski J , Ekelund E : Antibody binding to platelet antigens in acute and chronic idiopathic thrombocytopenic purpura: a platelet membrane ELISA for the detection of antiplatelet antibodies in serum. Clin Exp Immunol 1986; 63:459–65 3698340 PMC1577386

[35] Berchtold P , McMillan R , Tani P , *et al*: Autoantibodies against platelet membrane glycoproteins in children with acute and chronic immune thrombocytopenic purpura. Blood 1989; 74:1600–2 2790189

[36] Imbach P , Wagner HP , Berchtold W , *et al*: Intravenous immunoglobulin versus oral corticosteroids in acute immune thrombocytopenic purpura in childhood. Lancet 1985; 2:464–8 2863492 10.1016/s0140-6736(85)90400-3

[37] Nielsen OH , Tuckuviene R , Nielsen KR , *et al*: Flow cytometric measurement of platelet‐associated immunoglobulin in children with newly diagnosed Immune Thrombocytopenia. Eur J Haematol 2016; 96:397–403 26111053 10.1111/ejh.12605

[38] Debré M , Bonnet MC , Fridman WH , *et al*: Infusion of Fc gamma fragments for treatment of children with acute immune thrombocytopenic purpura. Lancet 1993; 342:945–9 8105212 10.1016/0140-6736(93)92000-j

[39] Iyori H , Fujisawa K , Akatsuka J : Autoantibodies and CD5+ B cells in childhood onset immune thrombocytopenic purpura. Acta Paediatr Jpn 1995; 37:325–30 7645381 10.1111/j.1442-200x.1995.tb03323.x

[40] Imbach P , Tani P , Berchtold W , *et al*: Different forms of chronic childhood thrombocytopenic purpura defined by antiplatelet autoantibodies. J Pediatr 1991; 118(4 Pt 1):535–9 2007927 10.1016/s0022-3476(05)83373-6

[41] Winiarski J : IgG and IgM antibodies to platelet membrane glycoprotein antigens in acute childhood idiopathic thrombocytopenic purpura. Br J Haematol 1989; 73:88–92 2803983 10.1111/j.1365-2141.1989.tb00225.x

[42] Biglino P , Perutelli P , Mori PG : Circulating antiplatelet antibody specificity in children with immune thrombocytopenic purpura at onset. Haematologica 1997; 82:127 9107104

[43] Fu L , Cheng Z , Gu H , *et al*: Platelet‐specific antibodies and differences in their expression in childhood immune thrombocytopenic purpura predicts clinical prognosis. Pediatr Invest 2018; 2:230–5 10.1002/ped4.12097 PMC7331365 32851271

[44] Deeks JJ , Altman DG : Diagnostic tests 4: likelihood ratios. BMJ 2004; 329:168–9 15258077 10.1136/bmj.329.7458.168 PMC478236

[45] Taaning E , Petersen S : Pattern of platelet‐associated immunoglobulin (classes and IgG subclasses) in childhood immune thrombocytopenic purpura. Eur J Haematol 1988; 41:449–53 3208868 10.1111/j.1600-0609.1988.tb00226.x

[46] Brink S , Hesseling PB , Amadhila S , *et al*: Platelet antibodies in immune thrombocytopenic purpura and onyalai. S Afr Med J 1981; 59:855–8 7015533

[47] Nielsen HE , Andersen EA , Carlsen N , *et al*: Presence of platelet antibodies in idiopathic thrombocytopenic purpura may discriminate acute from chronic disease. Acta Paediatr 2003; 92:1208–10 14632340

[48] Biglino P , Perutelli P , Mori PG : Platelet antibody detection in pediatric immune thrombocytopenic purpura: evaluation of three screening methods. Vox Sang 1997; 72:242–6 9228716 10.1046/j.1423-0410.1997.7240242.x

[49] Nieminen U , Peltola H , Syrjala MT , *et al*: Acute thrombocytopenic purpura following measles, mumps and rubella vaccination. A report on 23 patients. Acta Paediatr 1993; 82:267–70 8495082 10.1111/j.1651-2227.1993.tb12657.x

[50] Heitink‐Polle KMJ , Nijsten J , Boonacker CWB , *et al*: Clinical and laboratory predictors of chronic immune thrombocytopenia in children: a systematic review and meta‐analysis. Blood 2014; 124:3295–307 25305206 10.1182/blood-2014-04-570127

