# Supplementary material for: Anti‐platelet antibody immunoassays in childhood immune thrombocytopenia: a systematic review
Source: Vox Sang. 2020 Feb 20;115(4):323–33. doi: 10.1111/vox.12894 (PMC7317748; doi:10.1111/vox.12894)
Supplement: Supplementary file 1 — Fig. S1 Quality assessment of all 40 included studies, with judgement presented per study. [file VOX-115-323-s001.pdf]

Online Supplement

Anti-Platelet Antibody Immunoassays in Childhood Immune Thrombocytopenia: A Systematic Review

D.E. Schmidt, A.J. Lakerveld, K.M.J. Heitink-Pollé, M.C.A. Bruin,

G. Vidarsson, L. Porcelijn, M. de Haas

Supplementary Figure S1. Quality assessment of all 40 included studies, with judgement presented per study. Assessment was performed using the Quality Assessment for Diagnostic Accuracy Studies (QUADAS2) tool.

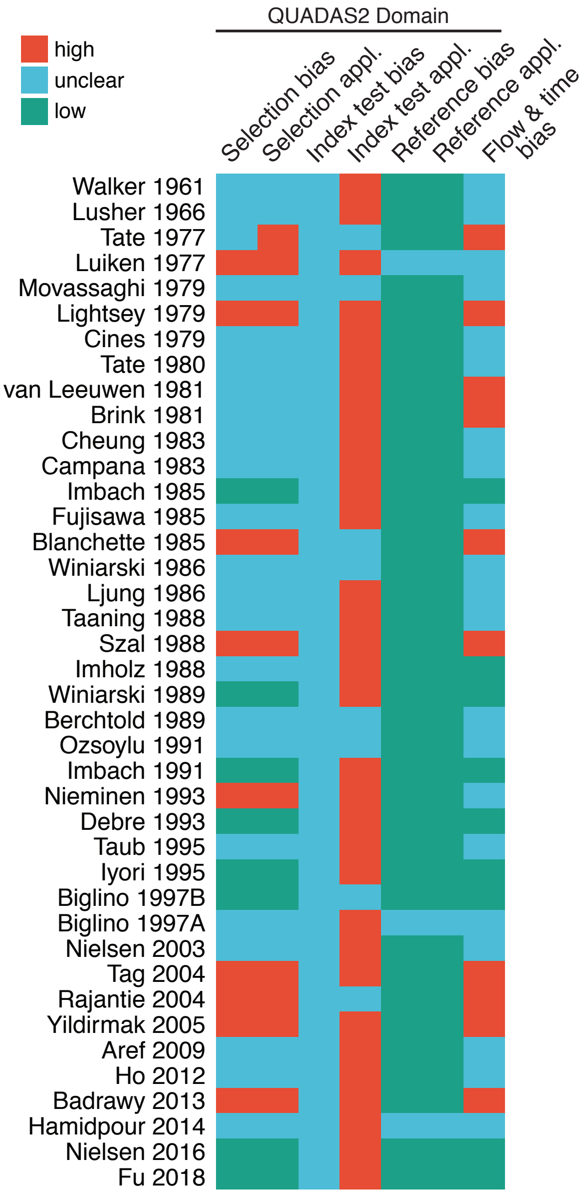

Appl., applicability concerns.
Bias, risk of bias.

Supplementary Methods

Study identification and screening

This systematic review follows reporting standards outlined in the PRISMA guidelines. It has not been pre-registered. A search strategy was developed for the PubMed and EMBASE databases to determine the diagnostic accuracy of various immunoassays measuring platelet autoantibodies in childhood ITP (Supplementary Table S1). In brief, the search string contained three elements: domain (children between 3 months and 18 years old), disease (ITP), and diagnostic tests for comparison (platelet autoantibody tests). Databases were searched for all articles indexed from inception until April 4, 2019. Studies assessing all various disease states (newly diagnosed, chronic) were included. Congress abstracts were excluded in EMBASE. The sensitivity of the search strategy was assessed by the inclusion of pre-determined index publications. Screening of abstracts was performed independently by two investigators (D.S. and A.L.). All selected studies were reviewed in full text.

Study eligibility, data extraction and validation

After initial screening, articles were assessed for eligibility in full-text by the same two investigators. Studies were included if they were published in English language and evaluated a platelet autoantibody assay in children with immune thrombocytopenia, irrespective of the definition of diagnosis or the duration of disease. The anti-platelet antibody immunoassay needed to be previously established in a peer-reviewed publication, or sufficiently described in the manuscript. Studies were excluded if they did not separate adult and childhood ITP data, assessed neonates or used anti-platelet antibodies as criteria for the diagnosis of ITP. Review articles, case reports, and series (less than ten patients) were excluded. The following data was extracted from included studies using standardized data collection forms:

characteristics of study population (setting, sample size, age, gender, chronic ITP rate, preceding infection rate, bleeding symptoms, treatments, type and number of controls), and characteristics of tests under evaluation (assay, number of positives and negatives for ITP patients and controls). Data was extracted separately per disease stage if it was presented in such a way. When data for heterogeneous populations (concerning disease stage or treatment) was given, but a subset was specified for a group of `acute onset` or `untreated`, the data of this subset of patients were extracted (as indicated). Data collection was validated independently by a third investigator (L.P.). For all included studies we calculated sensitivity and specificity as well as positive and negative likelihood ratios [1,2]. These measures of diagnostic accuracy are not influenced by the ratio of number of controls and cases, making them useful in studies where investigators determined this ratio.

Definitions

The original terminology of the authors was kept for `acute` and `chronic` ITP. We used the term `at diagnosis` exclusively for patients that had recently been diagnosed, i.e. within one week, and referred to `transient ITP` when patients had resolution of disease within three months [3]. `Direct` and `indirect` tests were defined as the measurement of antibodies bound to autologous patient platelets (direct) or measurement of circulating autoantibodies in either serum or plasma (indirect).

Immunoassay classes were defined by assay principle into enzyme-linked immunosorbent assay (ELISA), platelet-associated immunoglobulin (PA-Ig) [4-6], platelet immunofluorescence technique (PIFT) [7], monoclonal antibody-specific immobilization of platelet antigens (MAIPA) [8], immunoblot and immunobead or functional assays.

Assessment of methodological quality

The methodological quality of included articles was assessed using a standardized protocol for quality assessment of diagnostic accuracy studies (QUADAS2) [9], after tailoring to the specific research question of this systematic review and developing review-specific guidance for judgment. The questionnaire was initially tested on a random sample of four articles, subsequently refined and then applied to all articles. Publication bias was not assessed.

Supplementary References

1. Knottnerus AJ, editor. Evidence Base of Clinical Diagnosis. London : BMJ Books; 2002.
2. Deeks JJ, Altman DG. Diagnostic tests 4: likelihood ratios. BMJ. 2004 Jul 17;329(7458):168–9.
3. Rodeghiero F, Stasi R, Gernsheimer T, Michel M, Provan D, Arnold DM, et al. Standardization of terminology, definitions and outcome criteria in immune thrombocytopenic purpura of adults and children: report from an international working group. Blood. American Society of Hematology; 2009 Mar 12;113(11):2386–93.
4. Dixon R, Rosse W, Ebbert L. Quantitative determination of antibody in idiopathic thrombocytopenic purpura. Correlation of serum and platelet-bound antibody with clinical response. New England Journal of Medicine. 1975 Jan 30;292(5):230–6.
5. McMillan R, Smith RS, Longmire RL, Yelenosky R, Reid RT, Craddock CG. Immunoglobulins associated with human platelets. Blood. 1971 Mar;37(3):316–22.
6. Luiken GA, McMillan R, Lightsey AL, Gordon P, Zevely S, Schulman I, et al. Platelet-associated IgG in immune thrombocytopenic purpura. Blood. 1977 Aug;50(2):317–25.
7. Borne von dem AEGK, Verheugt FWA, Oosterhof F, Riesz E, Rivière AB, Engelfriet CP. A Simple Immunofluorescence Test for the Detection of Platelet Antibodies. British Journal of Haematology. Blackwell Publishing Ltd; 1978 Jun;39(2):195–207.
8. Kiefel V, Santoso S, Weisheit M, Mueller-Eckhardt C. Monoclonal antibody--specific immobilization of platelet antigens (MAIPA): a new tool for the identification of platelet-reactive antibodies. Blood. 1987 Dec;70(6):1722–6.
9. Whiting PF, Rutjes AWS, Westwood ME, Mallett S, Deeks JJ, Reitsma JB, et al. QUADAS-2: a revised tool for the quality assessment of diagnostic accuracy studies. Ann Intern Med. 2011 Oct 18;155(8):529–36.

Full references to the included studies

Walker, J. H., & Walker, W. (1961). Idiopathic thrombocytopenic purpura in childhood. Archives of Disease in Childhood, 36, 649–657.

Lusher, J. M., & Zuelzer, W. W. (1966). Idiopathic thrombocytopenic purpura in childhood. The Journal of Pediatrics, 68(6), 971–979.

Luiken, G. A., McMillan, R., Lightsey, A. L., Gordon, P., Zevely, S., Schulman, I., et al. (1977). Platelet-associated IgG in immune thrombocytopenic purpura. Blood, 50(2), 317–325.

Tate, D. Y., Sorenson, R. L., Gerrard, J. M., White, J. G., & Krivit, W. (1977). An immunoenzyme histochemical technique for the detection of platelet antibodies from the serum of patients with idiopathic (autoimmune) thrombocytopenic purpura (ITP). British Journal of Haematology, 37(2), 265–275.

Cines, D. B., & Schreiber, A. D. (1979). Immune thrombocytopenia. Use of a Coombs antiglobulin test to detect IgG and C3 on platelets. New England Journal of Medicine, 300(3), 106–111. http://doi.org/10.1056/NEJM197901183000302

Lightsey, A. L. J., Koenig, H. M., McMillan, R., & Stone, J. R. J. (1979). Platelet-associated immunoglobulin G in childhood idiopathic thrombocytopenic purpura. The Journal of Pediatrics, 94(2), 201–204.

Movassaghi, N., Moorhead, J., & Leikin, S. (1979). Anti-platelet antibodies in childhood idiopathic thrombocytopenic purpura. American Journal of Diseases of Children (1960), 133(3), 257–259.

Tate, D. Y., Carlton, G. T., Nesbit, M. E., White, J. G., Krivit, W., & Sorenson, R. L. (1980). Detection of platelet associated IgG in immune thrombocytopenia: a new assay employing protein A and peroxidase anti-peroxidase (PROA-PAP). American Journal of Hematology, 9(4), 349–361.

Brink, S., Hesseling, P. B., Amadhila, S., & Visser, H. S. (1981). Platelet antibodies in immune thrombocytopenic purpura and onyalai. South African Medical Journal = Suid-Afrikaanse Tydskrif Vir Geneeskunde, 59(24), 855–858.

van Leeuwen, E. F., Borne, von dem, A. E., van der Plas-van Dalen, C., & Engelfriet, C. P. (1981). Idiopathic thrombocytopenic purpura in children; detection of platelet autoantibodies by immunofluorescence. Scandinavian Journal of Haematology, 26(4), 285–291. http://doi.org/10.1111/j.1600-0609.1981.tb01663.x

Campana, D., Bergui, L., Camussi, G., Miniero, R., Morgando, M. P., Sardi, A., et al. (1983). Immune-complexes and anti-platelet antibodies in idiopathic thrombocytopenic purpura. Haematologica, 68(2), 157–166.

Cheung, N. K., Hilgartner, M. W., Schulman, I., McFall, P., Glader, B. E., N K, C., V, et al. (1983). Platelet-associated immunoglobulin G in childhood idiopathic thrombocytopenic purpura. Journal of Pediatrics, 102(3), 366–370.

Blanchette, V., Hogan, V., Esseltine, D., Hsu, E., Luke, B., & Rock, G. (1985). Evaluation of a simple immunodiffusion technique for quantitation of platelet-associated immunoglobulin G in childhood immune thrombocytopenias. The American Journal of Pediatric Hematology/Oncology, 7(2), 125–131.

Fujisawa, K., & Akatsuka, I. J. (1985). Platelet-associated IgG in children. Values and evaluation of PAIgG in various thrombocytopenias. Acta Paediatrica Japonica : Overseas Edition, 27(3), 445–454.

Imbach, P., Wagner, H. P., Berchtold, W., Gaedicke, G., Hirt, A., Joller, P., et al. (1985). Intravenous immunoglobulin versus oral corticosteroids in acute immune thrombocytopenic purpura in childhood. Lancet (London, England), 2(8453), 464–468.

Ljung, R., Nilsson, I. M., Frohm, B., & Holmberg, L. (1986). Platelet-associated IgG in childhood idiopathic thrombocytopenic purpura: measurements on intact and solubilized platelets and after gammaglobulin treatment. Scandinavian Journal of Haematology, 36(4), 402–407.

Winiarski, J., & Ekelund, E. (1986). Antibody binding to platelet antigens in acute and chronic idiopathic thrombocytopenic purpura: a platelet membrane ELISA for the detection of anti-platelet antibodies in serum. Clinical and Experimental Immunology, 63(2), 459–465.

Imholz, B., Imbach, P., Baumgartner, C., Berchtold, W., Gaedicke, G., Gugler, E., et al. (1988). Intravenous immunoglobulin (i.v. IgG) for previously treated acute or for chronic idiopathic thrombocytopenic purpura (ITP) in childhood: A prospective multicenter study. Blut, 56(2), 63–68.

Szal, M., & Blumberg, N. (1988). Clinical correlates in patients with elevated platelet-associated immunoglobulins. Annals of Clinical and Laboratory Science, 18(1), 24–33.

Taaning, E., & Petersen, S. (1988). Pattern of platelet-associated immunoglobulin (classes and IgG subclasses) in childhood immune thrombocytopenic purpura. European Journal of Haematology, 41(5), 449–453.

Berchtold, P., McMillan, R., Tani, P., Sommerville-Nielsen, S., & Blanchette, V. S. (1989). Autoantibodies against platelet membrane glycoproteins in children with acute and chronic immune thrombocytopenic purpura. Blood, 74(5), 1600–1602.

Winiarski, J. (1989). IgG and IgM antibodies to platelet membrane glycoprotein antigens in acute childhood idiopathic thrombocytopenic purpura. British Journal of Haematology, 73(1), 88–92.

Imbach, P., Tani, P., Berchtold, W., Blanchette, V., Burek-Kozlowska, A., Gerber, H., et al. (1991). Different forms of chronic childhood thrombocytopenic purpura defined by anti-platelet autoantibodies. Journal of Pediatrics, 118(4 Pt 1), 535–539.

Ozsoylu, S., Karabent, A., Irken, G., & Tuncer, M. (1991). Anti-platelet antibodies in childhood idiopathic thrombocytopenic purpura. American Journal of Hematology, 36(2), 82–85.

Debré, M., Bonnet, M. C., Fridman, W. H., Carosella, E., Philippe, N., Reinert, P., et al. (1993). Infusion of Fc gamma fragments for treatment of children with acute immune thrombocytopenic purpura. Lancet (London, England), 342(8877), 945–949.

Nieminen, U., Peltola, H., Syrjala, M. T., Makipernaa, A., & Kekomäki, R. (1993). Acute thrombocytopenic purpura following measles, mumps and rubella vaccination. A report on 23 patients. Acta Paediatrica, 82(3), 267–270.

Iyori, H., Fujisawa, K., & Akatsuka, J. (1995). Autoantibodies and CD5+ B cells in childhood onset immune thrombocytopenic purpura. Acta Paediatrica Japonica : Overseas Edition, 37(3), 325–330.

Taub, J. W., Warrier, I., Holtkamp, C., Beardsley, D. S., & Lusher, J. M. (1995). Characterization of autoantibodies against the platelet glycoprotein antigens IIb/IIIa in childhood idiopathic thrombocytopenia purpura. American Journal of Hematology, 48(2), 104–107.

Biglino, P., Perutelli, P., & Mori, P. G. (1997[A]). Platelet antibody detection in pediatric immune thrombocytopenic purpura: evaluation of three screening methods. Vox Sanguinis, 72(4), 242–246.

Biglino, P., Perutelli, P., & Mori, P. G. (1997[B]). Circulating anti-platelet antibody specificity in children with immune thrombocytopenic purpura at onset. Haematologica, 82(1), 127.

Nielsen, H. E., Andersen, E. A., Carlsen, N., Nir, M., & Taaning, E. (2003). Presence of platelet antibodies in idiopathic thrombocytopenic purpura may discriminate acute from chronic disease. Acta Paediatrica, 92(10), 1208–1210.

Tag, L. M., Ezz-Eldeen, A. M., Mahmoud, M. S., Rashed, H.-A. G., Noaman, H. A., L M, T., et al. (2004). Serum IL-2 and platelet-associated immunoglobulins are good prognostic markers in immune thrombocytopenic purpura. The Egyptian Journal of Immunology, 11(2), 121–132.

Rajantie, J., Javela, K., Joutsi-Korhonen, L., & Kekomäki, R. (2004). Chronic thrombocytopenia of childhood: use of non-invasive methods in clinical evaluation. European Journal of Haematology, 72(4), 268–272. http://doi.org/10.1111/j.1600-0609.2004.00215.x

Yildirmak, Y., Yanikkaya-Demirel, G., Palanduz, A., & Kayaalp, N. (2005). Antiplatelet Antibodies and Their Correlation with Clinical Findings in Childhood Immune Thrombocytopenic Purpura. Acta Haematologica, 113(2), 109–112. http://doi.org/10.1159/000083448

Aref, S., Selim, T., Ibrahim, L., Abd-Elghaffar, H., & Ashery, R. E. (2009). Flow cytometry detection of platelets autoantibodies in children with idiopathic thrombocytopenic purpura. Indian Journal of Hematology & Blood Transfusion : an Official Journal of Indian Society of Hematology and Blood Transfusion, 25(3), 96–103. http://doi.org/10.1007/s12288-009-0028-0

Ho, W.-L., Lee, C.-C., Chen, C.-J., Lu, M.-Y., Hu, F.-C., Jou, S.-T., et al. (2012). Clinical features, prognostic factors, and their relationship with anti-platelet antibodies in children with immune thrombocytopenia. Journal of Pediatric Hematology/Oncology, 34(1), 6–12. http://doi.org/10.1097/MPH.0b013e3182282548

Badrawy, H., Elsayh, K. I., Zahran, A. M., El-Ghazali, M. H., H, B., K I, E., et al. (2013). Platelet antibodies, activated platelets and serum leptin in childhood immune thrombocytopenic purpura. Acta Haematologica, 130(4), 312–318. http://doi.org/http://dx.doi.org/10.1159/000353384

Hamidpour, M., Khalili, G., Tajic, N., Shamsian, B. B. S., Hamidpour, R., M, H., et al. (2014). Comparative of three methods (ELIZA, MAIPA and flow cytometry) to determine anti-platelet antibody in children with ITP. American Journal of Blood Research, 4(2), 86–92.

Nielsen, O. H., Tuckuviene, R., Nielsen, K. R., & Rosthøj, S. (2016). Flow cytometric measurement of platelet-associated immunoglobulin in children with newly diagnosed Immune Thrombocytopenia. European Journal of Haematology, 96(4), 397–403. http://doi.org/10.1111/ejh.12605

Fu, L., Cheng, Z., Gu, H., & Wu, R. (2018). Platelet-specific antibodies and differences in their expression in childhood immune thrombocytopenic purpura predicts clinical prognosis. Pediatric Investigation, 2(4), 230–235.
